# Routine analysis of ascitic fluid for evidence of infection in children with chronic liver disease: Is it mandatory?

**DOI:** 10.1371/journal.pone.0203808

**Published:** 2018-10-05

**Authors:** Carolyne Ghobrial, Engy Adel Mogahed, Hanaa El-Karaksy

**Affiliations:** Pediatric Hepatology Unit, Pediatrics Department, Kasr Alainy School of Medicine, Cairo, Egypt; Western Sydney University, AUSTRALIA

## Abstract

Ascitic fluid infection is a major cause of morbidity and mortality in cirrhotic patients, requiring early diagnosis and therapy. We aimed to determine predictors of ascitic fluid infection in children with chronic liver disease. The study included 45 children with chronic liver disease and ascites who underwent 66 paracentesis procedures. Full history taking and clinical examination of all patients were obtained including fever, abdominal pain and tenderness and respiratory distress. Investigations included: complete blood count, C-reactive protein, full liver function tests, ascitic fluid biochemical analysis, cell count and culture. Our results showed that patients’ ages ranged between 3 months to 12 years. Prevalence of ascitic fluid infection was 33.3%. Gram-positive bacteria were identified in six cases, and Gram-negative bacteria in five. Fever and abdominal pain were significantly more associated with infected ascites (p value = 0.004, 0.006). Patients with ascitic fluid infection had statistically significant elevated absolute neutrophilic count and C-reactive protein. Logistic regression analysis showed that fever, abdominal pain, elevated absolute neutrophilic count and positive C-reactive protein are independent predictors of ascitic fluid infection. Fever, elevated absolute neutrophilic count and positive C-reactive protein raise the probability of ascitic fluid infection by 3.88, 9.15 and 4.48 times respectively. The cut-off value for C-reactive protein for ascitic fluid infection was 7.2 with sensitivity 73% and specificity of 71%. In conclusion, prevalence of ascitic fluid infection in pediatric patients with chronic liver disease and ascites was 33.3%. Fever, abdominal pain, positive C-reactive protein and elevated absolute neutrophilic count are strong predictors of ascitic fluid infection. Therefore an empirical course of first-line antibiotics should be immediately started with presence of any of these predictors after performing ascitic fluid tapping for culture and sensitivity. In absence of these infection parameters, routine ascitic fluid analysis could be spared.

## Introduction

Ascites is a common problem in patients with chronic liver disease (CLD), which develops secondary to intrahepatic portal hypertension [1]. Those patients are particularly susceptible to infections with a higher prevalence in cirrhotics [2]. Liver dysfunction is known to impair defense mechanisms against infection because of depressed reticuloendothelial system phagocytic activity, reduced serum complement levels and low antibacterial activity of ascitic fluid [3].

Ascitic fluid infection (AFI) represents a major cause of morbidity and mortality in cirrhotic patients [2]. In the past, mortality rate ranged between 80–100% of cases [4,5]. With early diagnosis and prompt initiation of appropriate therapy, recent reports showed dramatic decline in mortality rates secondary to AFI compared to older studies [6,7]. In children, about 28–43% of liver disease-related ascites have AFI with 24% 1-year mortality [1,8].

AFI has been classified into three types based on the results of ascitic fluid polymorphonuclear (PMN) cell count and culture [4,6,9]: 1- Spontaneous bacterial peritonitis (SBP) defined as absolute count of PMN in ascitic fluid >250/mm^3^ with a single type of bacteria on culture, 2- Culture-negative neutrocytic ascites (CNNA): negative ascitic fluid culture with PMN count of >250/mm^3^ and 3- Mono-microbial non-neutrocytic bacterascites (MNBA): ascitic fluid culture positive for one type of bacteria with PMN count of <250/mm^3^.

Both the American [10] and European [11] guidelines recommend testing for cell count and ascitic fluid culture to exclude the presence of AFI in adults. Culture and sensitivity results can guide for the right antibiotic choice, as resistance to commonly prescribed antibiotics is common in such patients [12], although only 50 to 70% of patients with AFI have positive ascitic fluid cultures [13,14].

Cadranel et al. (2013) reported in a cohort of asymptomatic cirrhotic outpatients a low incidence of SBP; thus exploratory paracentesis could be avoided in such patients without a significant risk [15]. In children, any painful procedure is considered invasive and is usually performed under sedation and analgesia. Consequently, performing paracentesis in a small child with ascites but with otherwise fair general condition may be discouraged. An empirical course of first-line antibiotics such as third-generation cephalosporins, fluoroquinolones, or piperacillin/tazobactam may be started, though identifying the infectious agent could be missed [16].

There is paucity of pediatric literature on AFI. The aim of our study was to detect predictors of AFI to determine if routine paracentesis could be avoided in selected cases.

## Materials and methods

This cross sectional study was carried out at the Pediatric Hepatology Unit, Cairo University, Egypt, from January 2014 to May 2016. All patients were enrolled in the study after an informed consent was obtained from their parents/guardians. The study was approved by the Research Ethics Committee of Pediatric Department, Faculty of Medicine, Cairo University, Egypt. The research was carried out in accordance to the Helsinki Declaration.

The study included 45 hospitalized children presenting with ascites secondary to CLD who underwent paracentesis. The severity of the underlying liver disease was assessed according to the pediatric end-stage liver disease (PELD) score [17].

Patients were excluded if they had secondary peritonitis, chylous, pancreatic, tuberculous, or biliary ascites. Patients who received antibiotics within the preceding week and patients with another source of infection were excluded as well.

Patients were subjected to history taking and clinical examination including diagnosis of the original CLD, indication of paracentesis whether diagnostic or therapeutic and symptoms and signs of peritonitis as fever, abdominal pain and tenderness, vomiting, diarrhea, worsening of ascites, tachypnea, deepening of jaundice and encephalopathy.

Investigations done included: complete blood count, C-reactive protein (CRP), liver function tests and kidney functions. A ratio of the albumin concentration of simultaneously sampled serum and ascites was calculated (Serum-ascites albumin gradient [SAAG]).

Paracentesis was performed under aseptic technique. Ten ml of ascitic fluid were inoculated at bedside in a blood culture bottle; the remaining portion of the ascitic fluid was used for biochemical analysis and cytology. We categorized our patients into 2 groups, the first one included children with AFI and the second group with non-infected ascites. Both groups were compared as regards clinical and laboratory parameters.

### Statistical methods

Data were collected and tabulated. Statistical Package for Social Science (SPSS) program version 20 was used for data analysis. Mean and standard deviation (SD) or median and interquartile range (IQR) were estimates of quantitative data including age and laboratory results; while frequency and percentage were estimates of qualitative data as sex and clinical data. Differences were tested by Student’s paired and unpaired t-test, Mann-Whitney U test or Wilcoxon test for quantitative data. Categorical variables as fever, abdominal pain etc were compared between the two groups using Chi square/Fisher exact test. A two-sided P value <0.05 was considered statistically significant. The odds ratio (OR) and their 95% confidence interval (CI) were used to evaluate clinical efficacy. To determine the test performance for prediction of AFI, a receiver operator characteristic (ROC) curve was constructed and area under the ROC curve (AUROC) was calculated with the corresponding 95% CI. An AUROC of greater than 0.7 was considered indicative of a fair test.

## Results

The study included 45 children with CLD and ascites with a total of 66 paracentesis procedures performed (7 patients had done more than 1 paracentesis in a different period of time more than 3 weeks from the previous paracentesis). Twenty-five patients were males (55.6%). Median age of the patients (IQR) was 1 (2.6) year, ranging between 3 months to 12 years. Biliary atresia constituted the main cause of CLD among the study group (33.3%) followed by idiopathic neonatal hepatitis (16%) (Table 1).

**Table 1:** Etiology of chronic liver disease in the study group (n = 45).

Diagnosis	Number of patients	Percentage
Biliary atresia	15	33.4
Idiopathic neonatal hepatitis	7	15.7
Progressive familial intrahepatic cholestasis I and II	5	11.1
Hepatic venous outflow obstruction	5	11.1
Cryptogenic cirrhosis	5	11.1
Niemann-Pick disease	2	4.4
Wilson disease	2	4.4
Tyrosinemia	2	4.4
Congenital hepatic fibrosis	1	2.2
Autoimmune hepatitis	1	2.2

Twenty-five paracentesis procedures were therapeutic to relieve distressing ascites. None of the 25 patients who had therapeutic tapping procedures had fever, abdominal pain, elevated total leukocytic count (TLC) or elevated CRP and only one of them had elevated absolute neutrophilic count (ANC). The remaining 41 procedures were diagnostic for suspicion of infection. Fever was present in 20 patients (49%), 5 patients (12%) had abdominal pain or tenderness, 8 had leukocytosis (19.5%), 37 had elevated ANC (90%) and CRP was positive in 36 cases (87.8%). At time of enrollment, none of the patients had encephalopathy, gastrointestinal bleeding, vomiting, diarrhea or impaired renal functions.

According to the results of ascitic fluid cell count and culture, 22 out of the 66 procedures (33.3%) were AFI. SBP was observed in 6/22 patients (27.3%), CNNA in 11/22 (50%) cases and MNBA in 5/22 (22.7%) cases. Two out of the 25 therapeutic paracentesis had AFI, while in the remaining 19 patients, paracentesis was done for diagnosis of suspected infection. Ascitic fluid culture was positive in 11/22 cases with AFI (SBP+MNBA). Gram-positive bacteria were detected in 6 cases (2 cases with Methicillin-resistant *Satphylococcus aureus* and 1 case with each of *Staphylococcus aureus*, *Enterococcus faecalis*, *Acinetobacter*, *Streptococci viridians*), while Gram-negative bacteria were identified in five cases (*Escherichia coli* in 2 cases, *Klebsiella pneumonia* in 2 cases and *Pseudomonas aeruginosa* in 1 case).

Twenty-two paracentesis procedures were done in 16 patients with infected ascites and 44 paracentesis procedures done in 29 patients with non-infected ascites. There were no statistically significant differences in the infected patients compared with non-infected patients as regards PELD score (P = 0.71).

Both groups with AFI and non-infected ascites were compared as regards clinical data and blood tests (Table 2). Fever, abdominal pain or tenderness, elevated TLC, ANC and positive CRP were significantly more frequent in the group with AFI. These variables did not show any significant difference among the 3 types of AFI (SBP, CNNA, MNBA). Liver function tests and biochemical analysis of ascitic fluid were comparable in both groups.

**Table 2:** Comparison between patients with infected and non-infected ascitic fluid as regards clinical data, laboratory blood parameters and PELD score.

	Infected ascites (N = 22)	Non-infected ascites (N = 44)	P value
**Clinical presentations:**			
Fever; N (%)	11 (50%)	9 (20.5%)	0.025*
Worsening or distressing ascites; N (%)	18 (81.8%)	42 (95.4%)	0.069
Abdominal pain or tenderness; N (%)	5 (22.7%)	0 (0%)	0.006*
**Laboratory parameters**:			
TLC /mm^3^; median (IQR)	11300(5700–15000)	9300 (7600–12850)	0.069
Patients with elevated TLC for age; N (%)	4 (18.2%)	4 (9.1%)	0.28
ANC/mm^3^; median (IQR)	4859 (2782–7021)	4020 (3018–5478.5)	0.025*
Patients with elevated ANC for age; N (%)	19 (86.4%)	18 (40.9%)	0.003*
Positive CRP (>6); N (%)	16 (72.7%)	20 (45.5%)	0.035*
Total serum bilirubin (<1mg/dL); median (IQR)	14.6 (7.6–20.5)	10 (2.95–14.64)	0.079
Conjugated bilirubin (<0.2mg/dL); median (IQR)	7.35 (3.4–11)	5 (1–6.9)	0.088
ALT (<40 U/L); median (IQR)	82 (49–98)	47 (41–55.5)	0.229
AST (<40 U/L); median (IQR)	141 (81–190)	112.5 (72–241)	0.36
AP (<360 U/L); median (IQR)	617.5 (794–295)	621.5 (409.5–815)	0.338
GGT (<50 U/L); median (IQR)	141 (89–258)	182.5 (88.5–438)	0.334
Serum albumin (3.5-5g/dl); mean ± SD	2.76 ± 0.6	2.715 ± 0.7	0.384
INR; median (IQR)	1.3(1.2–1.9)	1.5 (1.2–1.9)	0.82
SAAG; mean ± SD	1.915 ± 0.563	2.057 ± 0.68	0.203
PELD score	18.5 (11.8–22.0)	18.5 (10.0–27.5)	0.71

ALT: alanine aminotransferase, ANC: absolute neutrophilic count, AP: alkaline phosphatase, AST: aspartate aminotransferase, CRP: C-reactive protein, GGT: gamma glutamyle transpeptidase, INR: international normalized ratio, IQR: interquartile range, N: number, PELD: pediatric end stage liver disease, SAAG: Serum-ascites albumin gradient, SD: standard deviation, TLC: total leukocytic count.

* p-value is significant.

Logistic regression analysis was done for statistically significant variables suggestive of infection including fever, abdominal pain or tenderness, elevated TLC, ANC and CRP (Table 3). It showed that fever, abdominal pain, elevated ANC and CRP were independent predictors for AFI. Fever, elevated ANC and CRP increased the probability of AFI 3.88, 9.15 and 4.48 times respectively (Table 3).

**Table 3:** Multivariate logistic regression analysis for positive variables suggestive of ascitic fluid infection.

Variables	Infected ascites (N = 22)	Non infected ascites (N = 44)	Odds ratio (95% CI)	P- value
**Fever:**			3.88 (1.279 to 11.816)	0.0166*
Yes; N (%)	11 (50)	9 (20.5)		
No; N (%)	11 (50)	35 (79.5)		
**Abdominal pain:**			NA	0.003*
Yes; N (%)	5 (22.7)	0		
No; N (%)	17 (77.3)	44 (100)		
**TLC for age:**			2.22 (0.50 to 9.9)	0.420
Elevated; N (%)	4 (18.2)	4 (9%)		
Normal; N (%)	18 (81.8)	40 (91%)		
**ANC for age:**			9.1481 (2.352 to 35.568)	0.0014*
Elevated; N (%)	19 (86.4)	18 (41)		
Normal; N (%)	3 (13.6)	26 (59)		
**CRP:**			4.48 (1.399 to 14.928)	0.0115*
Positive; N (%)	17 (77.3)	19 (43.2)		
Negative; N (%)	5 (22.7)	25 (56.8)		

Odds ratio could not be done for abdominal pain, as one cell contains zero number of patients.

ANC: absolute neutrophilic count, CI: confidence interval, CRP: C-reactive protein, N: number, NA: not applicable, TLC: total leukocytic count.

* p-value is significant

**Fig 1:**
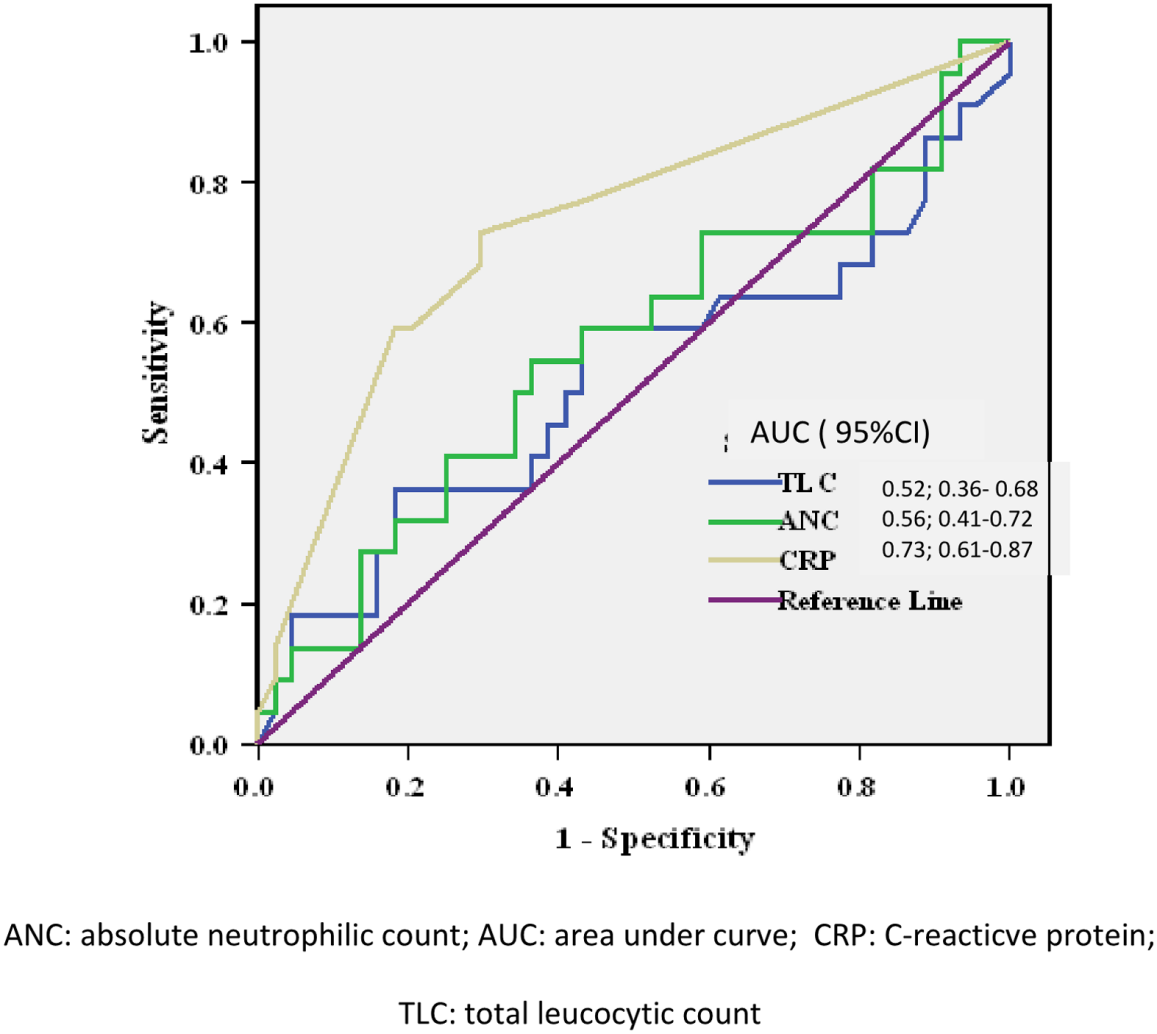
ROC curve for predictors of ascitic fluid infection.

In ROC curve analysis, CRP was the most significant predictor for AFI (p-value = 0.002 with AUROC: 0.73; 95% CI: 0.61–0.87). On the other hand, TLC and ANC were not significant predictors according to ROC curve. The cut-off value for CRP for AFI was 7.2, with sensitivity of 73% (95% CI: 0.54–0.91) and specificity of 71% (95% CI 0.57–0.84) (Fig 1).

## Discussion

AFI is one of the important causes of morbidity and mortality in CLD patients [18]. Early and proper management of these patients is essential to improve their outcome. Unfortunately, methods routinely used for diagnosis of AFI have a time limit ranging from few hours to days [19]. Extensive studies have been conducted to test efficiency of various markers for the diagnosis of AFI [8,20].

Pediatric literature on AFI is limited. Our main study question was whether routine ascitic fluid analysis is mandatory in suspected cases with infected ascites or are there other clinical and laboratory predictors of infection? Finding other alternative indicators for excluding AFI, sparing abdominal puncture in children would be of value taking into consideration that risk of AFI is very minimal in asymptomatic patients according to adult literature.

In our series, the prevalence of AFI was 33.3%. This was within the range reported in other studies conducted on children with suspected AFI with incidence ranging between 19–44% [21,22].

Fever is the most common symptom of AFI [1,23]. Moreover, abdominal pain and discomfort is one of the most frequent presenting complaints of AFI [23,24]. Similarly, in the current study, fever, abdominal pain and tenderness were significantly more frequent in pediatric patients with AFI. On the other hand, Srivastava et al. (2017) reported in their study that abdominal pain did not help in differentiating between patients with AFI and non-infected ascites. Although Srivastava et al. (2017) reported that 50% of patients with AFI could be asymptomatic; other studies report that asymptomatic patients have a very low or even null risk of AFI [1,15]. Similarly, in the current study only 2 out of the 25 patients who underwent therapeutic paracentesis, had AFI. It should be taken into consideration that respiratory distress in ascitic patients could be secondary to pain associated with AFI, not merely due to mechanical compression on the lungs.

In our study, ascitic fluid culture was negative in 50% of the cases with infected ascites. Ascites culture could be negative in up to 60% of patients with AFI [14,24]. The bacterial isolates in AFI are different in children versus adults, with Gram-negative organisms, mainly *Escherichia coli*, being most common in adults [24,25] and gram positive in children [26,27]. Our results revealed that gram-positive bacteria were slightly more prevalent than gram-negative isolates.

CRP may be used as a marker for early detection and monitoring of SBP in children with liver disease with high sensitivity and specificity [8,28]. Yuan et al. (2013) concluded that CRP is a better marker than TLC for diagnosis of patients with SBP [29]. These results were similar to what we found where positive CRP was a significant variable associated with SBP in both univariate and multivariate analysis and increases the probability of AFI by 4 times.

In our study, TLC in the peripheral blood per se showed no statistically significant difference between infected and non-infected ascites patients. Although, other studies found that TLC was significantly higher in patients with AFI [8,28]. We also analyzed ANC as a predictor of AFI and we found that it is a significant independent factor associated with ascitic infection. Elevated ANC raises probability of infected ascites 9 times more. This was contradictory to other reports done by Preto-Zamperlini et al. (2014) and Kalvandi et al. (2016) [8,28]. Discrepancy between the results of TLC and ANC as markers of ascitic infection, in our study, could be explained by the fact that patients with CLD usually have splenomegaly and hypersplenism that can mask the TLC elevation in peripheral blood.

One of our study limitations is the cross sectional nature of the study and the lack of serial measurement of CRP and ANC to detect their correlation with prognosis and treatment outcome.

In most laboratories, ascitic fluid cell count is done using manual techniques. This is time-consuming and liable to a high error rate and is not always accessible due to laboratory rush hours in referral hospitals or outpatient settings [22]. Although automated cell counting is faster, cheaper and more accurate, not all labs can provide automated cell counters for ascitic fluid since the manufacturers of this equipment do not recommend their use for counts on any fluid other than blood [30]. All the aforementioned methods for diagnosis of AFI need ascitic fluid tapping and analysis, which is more or less an invasive, painful procedure and will add to treatment costs especially in a developing country like Egypt. Our results revealed that presence of ≥2 of the following variables: fever, positive CRP and elevated ANC is considered a significant predictor of AFI. Thus, routine ascitic fluid analysis can be avoided in cases with less than 2 of these variables. Despite this conclusion, the relatively small sample size of children with infected ascitic fluid in our study is considered a study limitation that requires to be confirmed by a larger population study.

The decision to do abdominal tap for a child presenting with ascites might depend on local facilities of each institute. In centers with liver transplantation program receiving early referrals and having low wait-list mortality, an aggressive approach to any child with ascites may not be the first option. Organizational constraints may sometimes discourage to do a paracentesis in a small child with an otherwise unchanged general condition. The first approach in this case may start with evaluating the response to albumin and diuretics before moving on to perform an abdominal tap [31]. On contrary, Srivastava et al. (2017) concluded in their study on children with liver disease and ascites that 50% of cases with AFI had no clinical signs of infection at all and they recommended that all patients should be tapped and ascitic fluid should be analyzed for infection even in asymptomatic ones [1].

There was no significant difference in PELD score between patients with infected and non-infected ascites. These results are similar to a study done on hospitalized adult patients [32] and on outpatients with and without SBP [33].

The standard biochemical liver function tests were comparable in both groups of infected and non-infected ascites in our study. Other studies performed on pediatric patients with AFI reported similar results [21,26]. In contrast, Preto-Zamperlini et al. (2014) reported that serum albumin levels were significantly higher in the non-infected group while Kalvandi et al. (2016) found that serum albumin levels were significantly higher in the group with AFI [8,28]. The latter conclusion could be explained by that serum albumin level might be masked by previous albumin infusions in those patients. It should be put in consideration that serum albumin is a negative acute phase reactant.

The prevalence of AFI depends on severity of liver dysfunction, being higher in advanced liver disease [34]. High serum bilirubin is an important predictor for development of AFI in studies conducted on adults [5,25,35]. This finding was not observed in pediatric studies as elevated bilirubin is a common finding in many cholestatic liver diseases of infancy and childhood and is not necessarily associated with advanced liver disease as in adults. Cholestatic liver diseases in our study group constituted 60% of the cases.

In conclusion, prevalence of AFI in pediatric patients with CLD and ascites is 33.3%. Fever, abdominal pain, positive CRP and elevated ANC are strong predictors of AFI, therefore an empirical course of first-line antibiotics should be immediately started with presence of any of these predictors after performing ascitic fluid tapping for culture and sensitivity.

## Supporting information

S1 Table Supporting information for patients’ data. (XLSX)
